# 

**Published:** 1888-08-11

**Authors:** 


### WELCOME APPRECIATION AND THANKS.

I am directed by the President of the Metropolitan Hospital Sunday Fund to convey to the editor and proprietors of The Hospital annexed copy of resolution, passed unanimously by our Council at its last meeting.

Your obedient servant,

Mansion House, E.C. Henry N. Custance, Secretary.

Resolved unanimously: "That the cordial thanks of the Council be sent to the editor and proprietors of The Hospital for special exertions and publications in connection with the Hospital Sunday Fund, both before and after Hospital Sunday." By order,

Aug. 3rd. Henry N. Custance, Secretary.

### "The Hospital" Library of New and Standard Works.

For all information respecting the appearance of Titles, Prices, etc., of Books in this page, apply to Mr. W. H. Howe, office of The Hospital, 38, Old Jewry.

### Medical Library.

Abdomen; Diseases of the. By S. O. Habershon. 21/- *Churchill.*
Cancer: Its Allies, and other Tumours. By F. A. Purcell. 10/6 *Churchill.*
Diphtheria: Its Nature and Treatment. By Sir Morell Mackenzie. 5/- *Churchill.*
Ear: The Diseases of the. By A. Hartmann. 9/- *Pentland.*
Eye: On Diseases of the. By C. B. Taylor. *Redway.*
Heart: A Practical Treatise on Diseases of the. By W. H. Walshe. 16/- *Smith and Elder.*
Helps to Health. By Hy. C. Burdett. 1/6 *Kegan Paul.*
Help in Sickness. By Hy. C. Burdett. 1/6 *Kegan Paul.*
Liver: Diseases of the. By Geo. Harley. 21/- *Churchill.*
Lungs: A Practical Treatise on Diseases of the. By W. H. Walshe. 16/- *Smith and Elder.*
Midwifery for Midwives. A Manual of. By Fancourt Barnes. 6/- *Smith and Elder.*
Nervous System; A Manual of Diseases of the. By W. R. Gowers. 12/6 *Churchill.*

### Library of Biography and History.

Bacon. By R. W. Church (Dean of St. Paul's). 5/- *Macmillan.*

Gladstone; Anecdotes of. By an Oxford Man. 1/- *Hamilton.*

Greece: A History of. Part I. By Edwin Abbott. 10/6. *Rivingtons.*

Henry II. By Mrs. J. R. Green. 2/6. *Macmillan.*

Mitchell: Life of John. By Wm. Dillon. 2 vols. 21/- *Kegan Paul.*

### Library of Christian Literature.

A Century of Christian Progress and its Lessons. By J. Johnston. 3/- *Nisbet*
Faithful Departed; The Present State of the. By Rev. Geo. Body. 1/- *Masters.*
St. John, the Author of the Fourth Gospel. By H. H. Evans. 6/- *Nisbet.*
The Victory of the Cross. By B. F. Westcott. 3/6 *Macmillan.*

### Dictionaries and Encyclopædias.

Everybody's Pocket Cyclopædia. By E. Sheldon. 6d. *Saxon and Co.*
Middle English: A Concise Dictionary of. By A. L. Mayhew—W. W. Skeat. 7/6 *Froude.*
National Biography: Dictionary of. By Leslie Stephens 15/- *Smith and Elder.*
New English Dictionary on Historical Principles. Edited by James Murray. Part 4 (Bra-Cass). 12/6 *Froude.*

### Library of Fiction.

A Bitter Repentance. By Lady Virginia Sandars. 3 vols. *Hurst and Blackett.*
A Woman's Face. By F. Warden. 3 vols. *Ward and Downey.*
Children of Gibeon. By Walter Besant. 2/- *Chatto and Windus.*
Eve. A Romance. By Author of "John Herring." 2 vols. *Chatto and Windus.*
Jess. By H. Rider Haggard 2/6 *Smith and Elder.*

### To Secretaries and Matrons.

The Editor will be glad to have a copy of the Regulations, and also of every Report issued from the Press of Asylums, Hospitals, Convalescent and Nursing Institutions, as well as those of other Charities, so soon as they are published, and an early copy in duplicate of all appeals for funds. Notices of Annual and other Meetings, Festival Dinners, etc., will be welcomed. All such communications should be addressed to *The Editor, The Lodge, Porchester Square, London, W.* Any superintendent, secretary, matron, or other chief official who will regularly send such documents and information will be registered to receive free of cost a cover for binding The Hospital in half-yearly volumes on sending name and address to the publisher.

### Reversing the Order.

"Do you dance the lancers, Dr. Brown?"
"No, I do not dance the lancers;
But when the dancers' health breaks down
I sometimes lance the dancers."

In the Ionia House of Correction, in Michigan, the inmates wear a shoe with a heel of peculiar shape. Instead of cutting it straight across, it is made with a "diamond point." By the use of this device an escaped prisoner was recently tracked and recaptured; his trail was unmistakable.

### QUESTIONS AND ANSWERS.

E. D. has a friend who would like to establish a Convalescent Hospital for Children within twenty miles of London. She would be answerable for rent, taxes, and servants' wages, and has the promise of a permanent lady worker at £1 1s. per week; but she would like the promise of some help towards furnishing. She wishes to know also what would be a fair charge to make for each child per week. She proposes to dispense with recommendations, and to receive hip, spinal, surgical cases, as well as others. She thinks it possible that there might be a London Hospital to which a Convalescent Home of this sort might admirably be attached, cases which still require nursing being sent there for change when not eligible for ordinary homes.

### AMERICAN JOTTINGS.

A lady physician, Dr. Hattie Allen, has been appointed Associate Professor of Medicine in the University of Michigan. On this appointment the *Medical Record* says: "Now we hope that Hattie will write herself Harriet." We hope so too. These affectionate and playful contractions of Christian names, which abound and are appropriate in the domestic circle, are not in place in a larger sphere, any more than a childish lisp is becoming on a woman's lips, and they lose all their special charm when it is permitted to all and sundry to use them.

The amount of air required by man depends not only on size or bulk of each individual, but also to a great extent on age and exercise or work. The number of respirations at various ages may be thus tabulated: Infants, 44 respirations in each minute; 5 years of age, 26 respirations in each minute; 15 to 20 years of age, 20 respirations in each minute; 20 to 25 years of age, 18¾ respirations in each minute; 25 to 30 years of age, 16 respirations in each minute; 30 to 50 years of age, 18 respirations in each minute.

### The Student's Column.

### UNIVERSITY OF EDINBURGH.

The following is the official list of candidates who passed the second professional examination in July :—

F. R. B. Atkinson, Charles Ayres (B.A.), T. S. Balfour, J. C. H. Beaumont, J. H. Beilby, T. P. Blades, Richard Bland, A. S. Bowes (with distinction), E. A. Braithwaite, W. E. R. Branch, T. L. Bunting, T. J. Burton, C. S. R. Cadman, Donald Cameron, George Craig, S. G. Connor, William Currie, B. E. Dalison, Walter Dickson, D. G. Douglas, A. G. Dow, Henry Edwards, Matthew Elder, B. G. Ewing, Edward Fawcett, Gerald Fitzgerald (with distinction), James Fitzgerald, W. D. Forsyth, Thomas Frankish, R. C. Fryer, Alex. Gardner, A. B. Giles, P. H. Gillies, S. A. D Gillespie, W. E. Godber, E. M. Goldie, J Gordon-Munn, H. W. Greatbatch, A. E. H. C. Hallen, A. H. Hallen, T. R. Henderson, George Hennan (with distinction), H. G. Huie, T. W. Iddon, Hugh Jameson, Thomas Knowles, P. M. Kyle, H. G. Langwill (with distinction), G. E. Lanyon, William Lawton, O. R. Lewis, F. W. Lyle, S. G. M'Allum, D. F. M'Bean, A. J. M'Closky, W. S. M'Dougall, J. M. M'Gill (M.A.), Dugald M'Laren, J. L. Macrae, G. B. Marshall, F. A. Maynard, W. O. Meek, H. G. Melville (with distinction), A. E. Mole, G. H. Monro F. J. Nicoll. Maxwell Ogilvy-Ramsay, Owen Trafford Owen, J. C. Palmer, Walter Paterson, William Paterson (with distinction), E. A. Penfold, R. J. Pope (B.A.), A. F. M. Powell, J. R. M. Rae, R. R. Richardson, E. E. Roberts, J. D Robson, R. M. Ronaldson, F. W. F. Ross, Alexander Rutherford, A. F. Rutherford, A. O. Schorn, C. W. Scott, Robert Scott, R. P. Sharman, L. M. Silver (B.A.), W. V. Sinclair, J. W. Smith, W. C. Smith, James Spencer, W. C. W. Stain, T. P. Stewart, G. W. Thompson (with distinction), F. P. Trench, S. R. Webb, Abraham Wheeler, H. H. Wilde, E. M. Wilkes, A. F. Wilson, J. J. Wilson Richard Wyse.

### THE DEGREE OF DOCTOR OF MEDICINE, WITH THE TITLES OF THE THESES.

The following is the list of candidates who received the degree of Doctor of Medicine in the University of Edinburgh, on Wednesday, 1st August, 1888 :—

*Small Capitals indicate that the Candidate has passed the Examinations with First-Class Honours.*

*Italics indicate that the Candidate has passed the Examination with Second-Class Honours.*

*** Those who have obtained Gold Medals for their Dissertations.
** Deemed worthy of competing for the Dissertation Gold Medals
* Commended for their Dissertations.

Charles Aitken, India, M.B. C.M., 1883; Rachitis. James Auriol Armitage,* England (B.A.), M B., C.M. (with Second-Class Honours), 1886; Some Colouring Matters of Urine and their Pathological Significance. Edmund Kemp Bourne, England, M.B., C M., 1885; The Pathology and Treatment of Hæmorrhoids Herbert Bramwell, England, M.B., C.M., 1884; Fractures of the Base of the Skull. George Crichton. Scotland (M.A.), M.B, 1875; Anorexia: a Clinical Study. Alexander Henry Croucher, England, M.B., C.M., 1884; Inhalation of Anatomised Fluids, and its Application to Diseases of the Respiratory System. Thomas William Dewar,* Scotland, M.B., C.M., 1884; Some Functional Heart Murmurs; their probable Causation and Value as Diagnostic Signs. Andrew James Elliot,* Scotland, M.B., C.M., 1873; Remittent Fever as observed in Assam. David Griffith Evans, Anglesey, M.B, C M., 1883; Some Clinical Observations with Strophanthus hispidus. Richard Herbert Joseph Fetherston, Australia, M B. C.M., 1886; Laceration of the Cervix Uteri. Ormond Haldane Garland*, Scotland, M.B., C.M., 1868; Rheumatic Hyperpyrexia. Herbert James Gilbert, England, M.B., C.M., 1882; some Points in Dietetics. James Graham,*** Scotland (M.A.), M.B., C.M., 1882; a Clinical Study of Hydatid Disease. William Brendon Thompson Gubbin,* England, M.B., C.M., 1885; Biliousness and Lithæmia. Charles Henry Gwynn, England, M.B., C.M., 1878; a recent Medico-legal Case, with Notes and Experiments—Regina *v.* Forester. George Hall, England, M.B., C.M., 1886; Idiopathic Anæmia as occurring in Young Girls; its Causes, Pathology, Symptoms, and Treatment. John Berry Haycraft,*** England (B.Sc.), M.B., C.M., 1878; the History, Development, and Function of the Carapace of the Chelonia. *George Vickerman Hewlana*,** England, M B., C.M., 1885; Cocaine Toxæmia. Robert Stephen Hubbersty,* England, M.B., C.M., 1884; the Operative Treatment of Malignant Disease. James Hutcheson,* Scotland, M.B., C.M., 1886; Extra-Uterine Gestation. Frederick Miller Johnson,* Australia, M.B., C.M., 1886; Necrosis of the Cornea. Francis William Brandram Jones, England, M.B., C.M., 1883; Syphilis: its Course and Treatment. Charles Ashley Scott Leggatt, England, M.B., C.M., 1885; Psoriasis. Charles James Lewis, England, M.B., C.M. (with First-Class Honours), 1885; Diabetes Mellitus. William Maxwell Little, Singapore, M.B., C.M., 1885; Anæmia in Relation to Insanity. George Mackay,*** Madras, M.B., C.M. (with Second-Class Honours), 1883; Hemianopsia and Hemiachromatopsia. John M'Lachlan, Scotland, B.Sc., M.B., C.M., 1883; The Abortive Treatment of Specific (Idiopathic) Fevers. Murray MacLaren, New Brunswick (B.A.), M.B., C.M., 1884; Leprosy in New Brunswick. *John Maitland*,** India, M.B., C.M., 1874; Elephantoid Disease. *David James Mason*,** Scotland, M.B., C.M., 1885; The Excretion of Urea in Phthisis in its Relation to the Temperature and Blood. Angus Matheson, Scotland, M.B., C.M., 1884; Nasal Obstruction; its Etiology, Symptoms, and Treatment. Patrick William Maxwell,* Scotland. M.B., C.M., 1880; The Effect of Treatment on Strabismus. Thomas Cockburn Meggison,* England, M.B., C.M., 1882; The Methods of Diagnosis Employed in Diseases of the Thoracic Organs. William Francis Menzies, Canada, M.B, C.M., 1885; Body-Temperature of the Insane. *Alexander Cameron Miller*,** Scotland M.B., C.M., 1883; Insanity and the Neuroses. William Henry Miller,* Canary Islands, M.B., C.M., 1883; Obstetrical Statistics. Edwin Morton, England, M.B., C.M., 1885; electricity in the treatment of Disease. *James Musgrove*, ** England, M.B., C.N., 1886; Observations in Surgical Bacteriology. John Brady Nash, Australia, M.B., C M., 1882; Stone in the Bladder. David Thomson Playfair, Scotland, M.B., C.M., 1877. Some Difficulties in the Diagnosis of Chronic Rheumatism. Thomas Henry Pope, India, M.B., C.M, 1877; Tropical Abscesses of the Liver. John Billingsley Richardson, England, M.B., C.M., 1874; The Tubercular Form of Phthisis. Douglass M'Kissock Ross, England, M.B., 1874. Five Year's Experience of Poor-Law Medical and Surgical Practice. George Franklin Shiels, California, M.B., C.M., 1884; the Effects of Tobacco on those using it as a Luxury. George Thomson, Scotland, M.B., C.M. (with Second-Class Honours), 1884; Tubercular Disease of Bones and Joints: Clinical and Pathological Studies. Edmond Vaudrey, England, M.B., C.M., 1882; Fractures of the Femur. Norman Purvis Walker, **Scotland, M.B., C.M., 1884; Pleurisy: Clinical Notes and Remarks. *Thomas Boswall, Watson*, Scotland, M.B., C.M., 1872; Carcinoma of the mucous Surfaces, with Special Reference to the Œsophagus, Stomach, and Bladder.

### Amusements with Profit for all Ages.

### FOR MEN AND WOMEN.

**First Quarterly Poetical Competition.**

Commenced May 26th, 1888; ends August 25th, 1888.

This week credit will be given for

**No. 6.—Original Poem.**

THE RAINBOW.

When through the falling rain shines the bright sun,
Painting the drops in many a varied hue,
God's vow of promise, till this world be done,
Appears in ever-glowing colours to our view.
The fervent rose, the delicate softened green,
The golden tint of glory, earth-subdued,
Come to us freshly—in a new light seen,
Each time they come—with beauty fresh-imbued.

Speak gentle messenger of God's own sending—
Tell of His love and mercy never-ending;
Thus, though our way be ofttimes dark and dreary,
And when with vain-tried efforts we are weary,
Comes there a sudden light from Heaven above
Whence our great Father bendeth down in love,
Sending on earth's deep gloom his gladdening ray,
Making all darkness bright, turning all night to day.

K. M.

**Results No. 5.—Original Poem.**

Dodo (12), A. M. E. (12), Patience (12), Anthem (12), Hes perornis (10)

THE WORD COMPETITION.

**Results of Twelfth Week.**

*Lucerne.*—Dodo (19), Tinie (18) Patience (18), Lightowlers (18), Reldas (18), Puff (18), Sym (17), Lauriston (17), Alicia (11).

The above competition will be discontinued until after the Autumn, when New Prize Scheme will be started.

### THE CHILDREN'S CORNER.

PRIZES.
FOR MOST CORRECT ANSWERS TO PUZZLES.
This Commenced October 1st, 1887.
One Pound a Month.
A PRIZE OF THREE GUINEAS
to the Competitor who shall have sent in the largest number of correct or best answers during the twelve months.

**Puzzles Second Week.**

No. 5. Historical Mental Picture.—A young prince is passing through the courtyard of his father's palace, when his younger brothers, in a spirit of mischief, throw some dirty water over him. He being on bad terms with his father, regards this as a studied affront; and becomes so enraged that he draws his sword on them. This raises an uproar, which is not subdued till the king himself interferes. The prince, however, will not be satisfied, but retires from the palace. Give the names of the prince, his father, and brothers?

No. 6. Arithmetical Puzzle —In 1887 the product of the ages of a father and two sons was the number of the year. In what year was the elder born?

No. 7. Double Acrostic.—1. A town in Warwickshire. 2. A river in Spain. 3. Brief slumber. 4. A city of ancient Egypt. Initials give the name of a county, finals something for which it is celebrated.

No. 8. Enigma.

Two legs I have, but seldom walk on ground,
Yet when I run or move, one leg turns round.

**Results of Fourth Week Puzzles.**

No. 13. Divided Square.—1, 5, 7=13.

1 X 1=1.

5 X 5=25, difference 24.

7 X 7=49 „ 24.

Proof 1 + 25 + 49=75.

No. 14. Double Acrostic.

W iseacr E
A nn A
T umble R
E mme T
R ut H

No. 15. Enigma.—A Noun.

No. 16. Square Word.

S E A T
E L L A
A L M S
T A S K

**Results of Tenth Monthly Competition.**

This month we award the prize of £1 to Curly (Master M. Mason, Oswald Road, Oswestry, Shropshire). A. C. K. has gained a higher total, and Scrooge the same total of marks as Curly, but both are debarred from taking any share of the prize, through having already received one since October last.

**Notice to Correspondents.**

*Gem.*—Your total of marks is quite correct, no answers to second week's puzzles being received.

